# Prognostic value of epithelial–mesenchymal transition circulating tumor cells in female breast cancer: A meta-analysis

**DOI:** 10.3389/fonc.2022.1024783

**Published:** 2022-11-30

**Authors:** Qiang Zhao, Bingbing Li, Qi Gao, Yang Luo, Liang Ming

**Affiliations:** ^1^ Key Clinical Laboratory of Henan Province, Department of Clinical Laboratory, The First Affiliated Hospital of Zhengzhou University, Zhengzhou, China; ^2^ Henan Key Laboratory of Child Brain Injury and Henan Pediatric Clinical Research Center, Third Affiliated Hospital and Institute of Neuroscience, Zhengzhou University, Zhengzhou, China; ^3^ Center of Smart Laboratory and Molecular Medicine, Medical School, Chongqing University, Chongqing, China

**Keywords:** circulating tumor cells, epithelial-mesenchymal transition, prognostic value, breast cancer, meta-analysis

## Abstract

**Background:**

Epithelial–mesenchymal transition (EMT) conferred metastatic properties on circulating tumor cells (CTCs) and was considered to be correlated with bad survival outcomes in patients with breast cancer. However, different studies have reported controversial results regarding the relationship between CTCs that have undergone EMT (EMT-CTCs) and prognosis of breast cancer. Therefore, this meta-analysis aimed to investigate the prognostic role of EMT-CTCs in patients with breast cancer.

**Methods:**

In total, 842 patients from nine studies that were screened from Web of Science, Embase, and PubMed were included. The hazard ratio (HR) and 95% confidence interval (CI) for progression-free survival (PFS) and overall survival (OS) were extracted or estimated by the Kaplan–Meier survival curve for the meta-analysis. Sensitivity analysis was performed to characterize heterogeneity among the trials. Meanwhile, subgroup analysis was performed to present the effects of cancer stage, identification method, sampling volume, and region on the prognostic value of EMT-CTCs.

**Results:**

The pooled HRs for PFS were 1.97 (univariate: 95% CI, 1.19–3.24; *p* = 0.008) and 2.23 (multivariate: 95% CI, 1.29–3.86; *p* = 0.004). The pooled HRs for OS were 2.03 (univariate: 95% CI, 1.07–3.84; *p* = 0.029) and 1.70 (multivariate: 95% CI, 1.14–2.52; *p* = 0.009). Subgroup analysis showed that EMT-CTCs were associated with PFS in the primary breast cancer group (pooled HR = 2.58, 95% CI, 1.66–4.00, *p* < 0.001), the polymerase chain reaction (PCR) group (pooled HR = 2.69, 95% CI, 1.66–4.35, *p* < 0.001), the sampling volume of the >7.5-ml group (pooled HR = 1.93, 95% CI, 1.36–2.73, *p* < 0.001), and the Asia group (pooled HR = 1.92, 95% CI, 1.13–3.29, *p* = 0.017) and with OS in the primary breast cancer group (pooled HR = 3.59, 95% CI, 1.62–7.95; *p* = 0.002).

**Conclusion:**

The meta-analysis showed that EMT-CTCs were associated with poorer survival outcomes in patients with breast cancer. More accurate methods and designed clinical trials with unified standards are essential to establish the real role of EMT-CTCs in disease progression in women with breast cancer.

## 1 Introduction

By 2021, female breast cancer had become the most commonly diagnosed cancer worldwide (1). Although therapeutic treatment has improved, the incidence of the disease has increased conversely (2). Unfortunately, most patients are burdened with a poor prognosis, even those who have received treatment in the early stage (3). Invasion and metastasis were the main cause of death of cancer patients (4). Thus, available markers that could assist in expounding metastasis or predicting the patient’s prognosis are needed.

Circulating tumor cells (CTCs) were defined as tumor cells that had left their primary sites and entered peripheral blood (PB), leading to distal metastasis. Observed since the 19th century, CTCs had been considered as a promising tool in understanding cancer metastasis (5). In recent years, with the development of isolation and identification methods, attention to their clinical utility increased. The association between CTCs and survival outcome indicators, such as overall survival (OS) and progression-free survival (PFS), had been elaborated in many studies (6–8). Nevertheless, there were few current specifications or guidelines to clearly declare the prognostic value of CTCs in clinical practice (9). Insufficient data based on multicenter studies with large sample analysis might be one of the possible reasons. Furthermore, because the phenotype heterogenicity and small number in the bloodstream, it is a challenge to enumerate all CTCs without missing, which limited the accuracy of the CTCs analytic methods and might lead to controversial results among different trials.

Epithelial–mesenchymal transition (EMT) is an important phenomenon along with CTC circulation in PB. This phenomenon could upgrade the invasiveness of CTCs, making them more invasive, motile, and treatment-resistant (10). Some studies suggested that rare single cancer cells expressed mesenchymal or epithelial markers simultaneously while in its primary site (11). In contrast, once shed into the bloodstream, significant heterogeneity was observed. On the one hand, the transcription factors of EMT could be found in patients with early-stage cancer (12). On the other hand, expression of EMT markers in cancer cells in the primary site was not associated with those in CTCs (13). These results implied that EMT of CTCs occurred independently in the bloodstream, indicating its possible prediction value for patient outcome. Additionally, CTCs that have undergone EMT (EMT-CTCs) were associated with poorer outcomes in various epithelial malignancies, such as lung cancer (14, 15), colorectal cancer (16, 17), and head/neck cancer (18). In breast cancer, this combination has been observed in animal and clinical experiments (19, 20). However, because of the limited number of patients and inconsistent results of these studies, the relationship between EMT-CTCs and prognosis of breast cancer was controversial, limiting the study of the metastasis mechanism and the clinical application of EMT-CTCs. Therefore, we performed this meta-analysis with various related clinical studies to investigate the prognostic role of EMT-CTCs in patients with breast cancer.

## 2 Methods

### 2.1 Search strategy

In this meta-analysis, we searched studies from the databases of “Web of Science”, “Embase”, and “PubMed”. The mesh terms “epithelial–mesenchymal transition”, “breast neoplasms”, “circulating tumor cells”, and “prognosis” were searched with free terms through a combined strategy. To avoid omission of any qualified references during the database search, manual search was also performed.

### 2.2 Inclusion and exclusion criteria

The articles were included based on the following criteria (1): female breast cancer patients (2); EMT phenotypes were sorted from overall CTCs (3); prospective studies or retrospective ones with prognosis evaluation; and (4) the effect of EMT-CTCs on the survival was evaluated. No matter what the identification method was, a clear criterion should be presented to distinguish EMT-CTCs from other phenotypes. If more than one article investigated the same original population by the same study team, the one with the latest or most sufficient data was included. Otherwise, the articles with insufficient data or unavailable information like reviews, notes, meeting abstracts, chapters, case reports, and editorials were excluded.

### 2.3 Data extraction and quality assessment

The extracted data from eligible articles included first author name, time of publication, patients’ number, cancer stage, median age, detection method, region of study patients, and survival data involving HRs with 95% CI for PFS and/or OS. Data from univariate and multivariate subgroups were analyzed. For one study, HR was unavailable in full text and estimated from the presented Kaplan–Meier survival curves according to the method of Tierney et al. (21). Quality assessment of the included studies was performed by using the quality in prognosis studies (QUIPS) tool. This tool was used to assess the risk of bias in prognostic studies. A total of six bias domains were examined (1): study participation (2), study attrition (3), prognostic factor measurement (4), outcome measurement (5), study confounding, and (6) statistical analysis and reporting. Two reviewers evaluated the included studies independently, and disagreements were resolved by a third one. If any domain was classified as high risk, the study would be rated as high risk. When less than or equal to two moderate risks were considered, the paper was rated as low risk. Otherwise, the papers were classified as moderate risk.

### 2.4 Statistical analysis

Stata 12.1 was used for the meta-analysis. The heterogeneity between articles was evaluated by Cochran *Q* and the *I* ^2^ statistic. It was considered as statistically significant when *p* < 0.05 in the Cochran *Q* test. For the *I* ^2^ statistic, if *I* ^2^ > 50%, a random-effects model was used; otherwise, the fixed-effects model was applied. A random-effects model was used in the subgroup analysis. Moreover, sensitive analysis was performed to find the source of heterogeneity with the leave-one-out approach. The subgroup analysis was performed to assess the effect of several baseline characteristics on the prognosis value of EMT-CTCs. The publication bias was evaluated by Begg’s funnel plots and the trim-and-fill method.

## 3 Results

### 3.1 Study search

**Figure 1:**
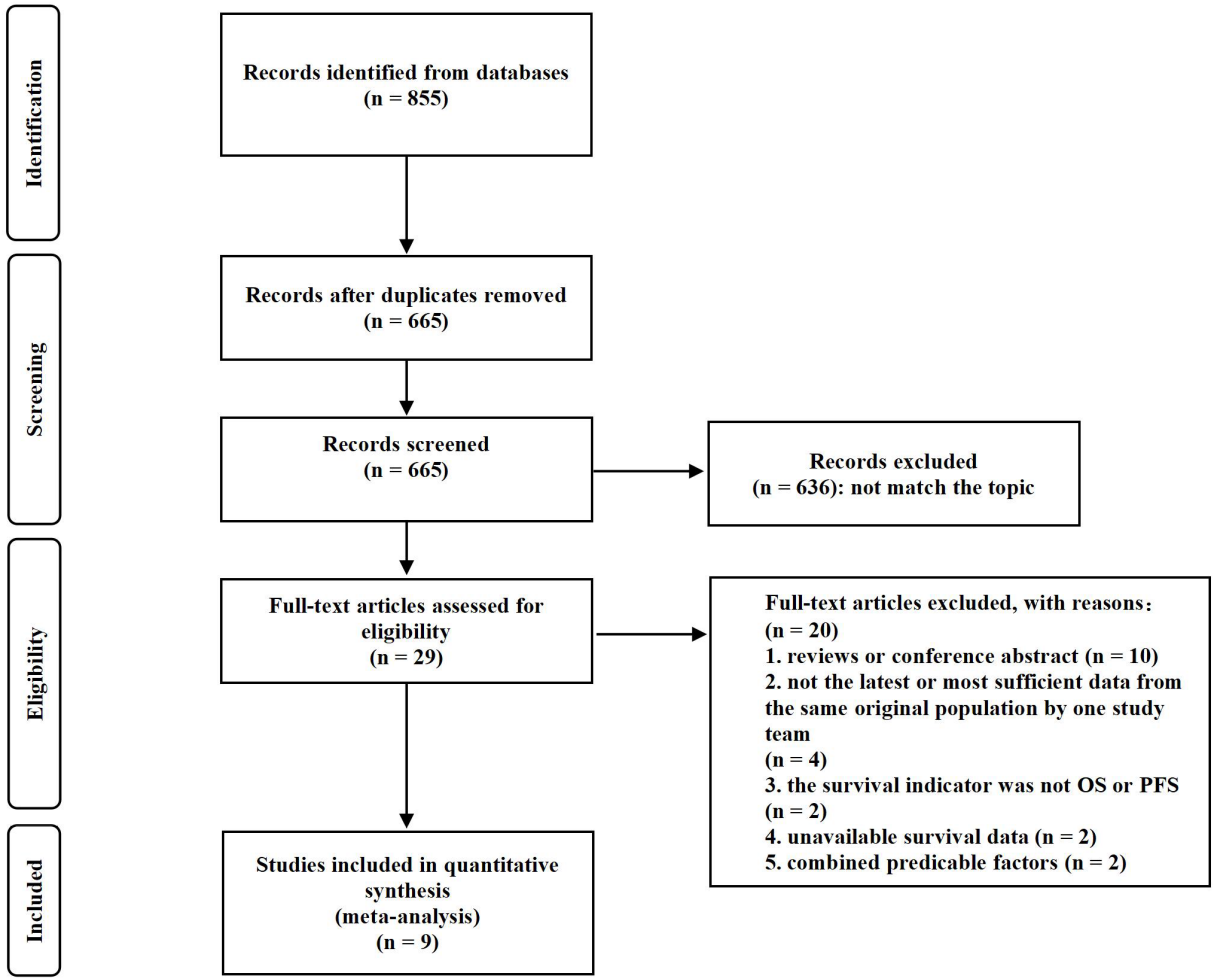
Flow diagram of study selection.

As shown in **Figure 1**, 855 results were obtained from the search procedure. After eliminating duplicates, 665 articles were interrogated. Then, 636 articles were excluded because their content did not match the topic and the other 29 documents were further interrogated. Among them, 20 documents were excluded due to the following reasons (1): reviews or conference abstracts (*n* = 10) (2); not the latest or most sufficient data from the same original population by the same study team (*n* = 4) (3); the survival indicator was not OS or PFS (*n* = 2) (4); unavailable survival data (*n* = 2); and (5) combined predicable factors (*n* = 2). Finally, nine studies were included for the meta-analysis.

### 3.2 Study characteristics and included data

Following the screening process, nine studies with a total of 842 patients were included for the meta-analysis. The detailed information is listed in **Table 1**. Four studies were conducted in Asia and Europe and one in North America. Although half of the articles focused on the patients with metastatic breast cancer, most of the patients (520/795) came from the studies with primary breast cancer. For the CTC detection method, five studies identified isolated CTCs with PCR-related methods based on the transcription factors of EMT markers, which involved Twist1, Vimentin, Snail, Slug, and so on. One study collected CTCs from patients’ apheresis sample. Moreover, Tan’s presented micRNA-106b as a novel marker to characterize EMT-CTCs.

**Table 1:** The detailed information of the included studies.

Author (year)	Cancer stage	Median age	Identification method	Sampling volume	Region	Number	EMT Marker	EMT-CTCs positivecriteria	Outcome	Reference
Guan (2019)	Metastatic Breast Cancer	51 (32–73)	Multi-RNA-ISH	5 ml	Asia	90	Vimentin, Twist1	Vimentin and Twist1 positive	PFS	(22)
Horimoto (2018)	Metastatic Breast Cancer	57 (38–81)	Immunostaining	10 ml	Asia	20	Vimentin	Vimentin positive	PFS	(23)
Mego (2012)	Metastatic Breast Cancer	44 (30–60)	PCR	7.5 ml	North America	19	Twist1, Snail	TF overexpression	PFS, OS	(24)
Miklikova (2020)	Primary Breast Cancer	59.1 (24.7–83.5)	PCR	9 ml	Europe	284	Twist1, Snail, Zeb1	TF overexpression	PFS	(25)
Tan (2019)	Metastatic Breast Cancer	—	PCR	7.5 ml	Asia	128	Vimentin, micRNA-106b	TF overexpression	OS	(26)
Markiewicz (2019)	Primary Breast Cancer	61.9 (39.1–82.6)	PCR	5 ml	Europe	72	Vimentin	TF overexpression	OS	(27)
Strati (2019)	Primary Breast Cancer	—	PCR	20 ml	Europe	100	Twist1	TF overexpression	PFS, OS	(28)
Chen (2020)	Primary Breast Cancer	50 (28–73)	Multi-RNA-ISH	5 ml	Asia	64	Vimentin, Twist1	Vimentin and Twist1 positive	PFS	(29)
Bulfoni (2016)	Metastatic Breast Cancer	62 (36–82)	Immunostaining	7.5 ml	Europe	47	CD44, CD146	CD44 and CD146 positive	PFS, OS	(30)

PFS, progression-free survival; OS, overall survival; TF, transcription factors.

The EMT-CTCs referred to the hybrid and/or mesenchymal phenotypes. Three of the studies separated them into two groups and reported HRs. We estimated pooled HRs with a random-effects model as previously reported (58). Bulfoni and colleagues calculated OS values for both stage IV diagnosis and the initial CTC assessment, and PFS values for the initial CTC assessment only. To correspond with other documents, the survival data were calculated since the initial CTC assessment was chosen.

### 3.3 Quality assessment

The results of the quality assessment analysis are shown in **Table 2**. It could be seen that three studies had low bias; the rest had moderate bias. The risk of bias might mainly come from study participation and attrition.

**Table 2:** The risk rating of the included studies.

Author (year)	Study participation	Study attrition	Prognostic factor measurement	Outcome measurement	Study confounding	Statistical analysis and reporting	Overall
Guan (2019)	Moderate	Moderate	Low	Low	Low	Low	Low
Horimoto (2018)	Moderate	Low	Low	Moderate	Moderate	Low	Moderate
Mego (2012)	Moderate	Moderate	Low	Low	Low	Low	Moderate
Miklikova (2020)	Low	Moderate	Low	Low	Low	Low	Low
Tan (2019)	Moderate	Moderate	Low	Low	Low	Low	Low
Markiewicz (2019)	Moderate	Moderate	Low	Low	Low	Low	Moderate
Strati (2019)	Moderate	Moderate	Low	Moderate	Low	Low	Moderate
Chen (2020)	Moderate	Moderate	Low	Low	Low	Low	Moderate
Bulfoni (2016)	Moderate	Moderate	Moderate	Low	Low	Low	Moderate

### 3.4 Pooled HRs for PFS and OS

**Figure 2:**
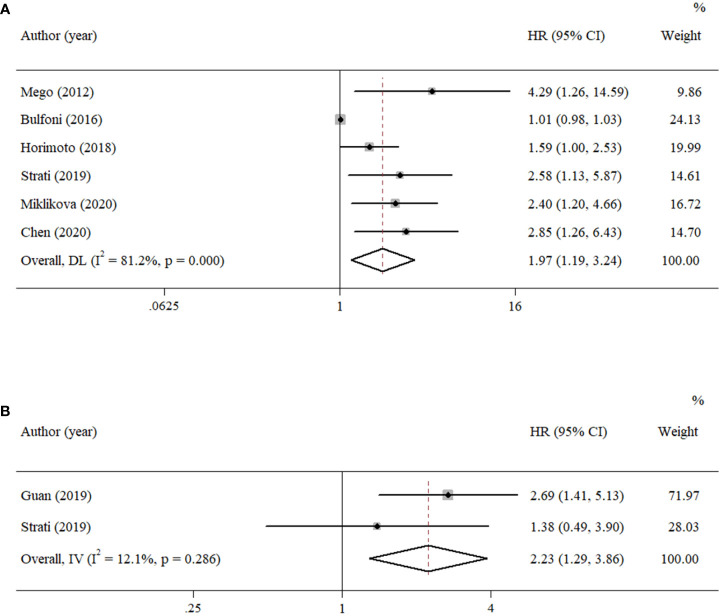
Forest plots of the pooled HRs for PFS with **(A)** univariate analysis and **(B)** multivariate analysis.

**Figure 3:**
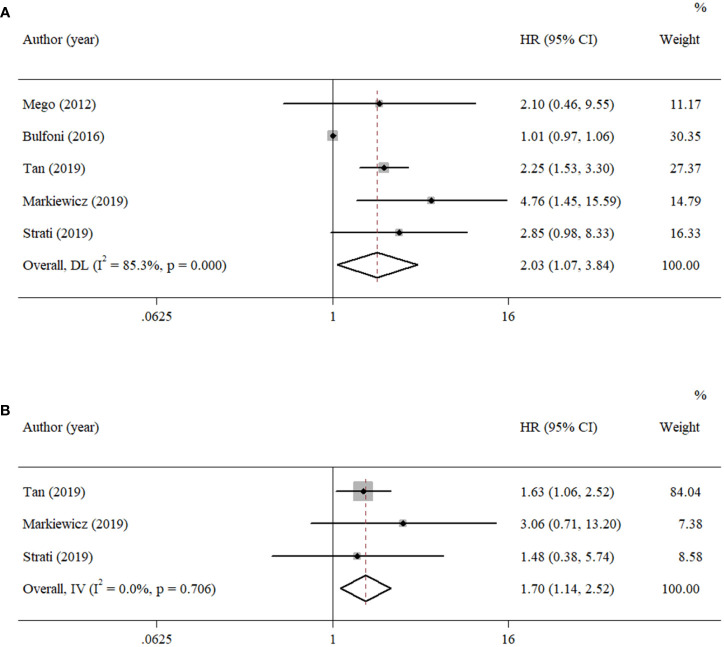
Forest plots of the pooled HRs for OS with **(A)** univariate analysis and **(B)** multivariate analysis.

According to the data from eligible documents, the pooled HRs for PFS and OS were calculated according to the multivariate and univariate groups, respectively. When significant heterogeneity was obtained (*I* ^2^ ≥ 50%), a random-effects model was performed; otherwise, a fixed-effects model was carried out. Six studies reported PFS with univariate analysis, and two studies reported PFS with multivariate analysis. Hence, the pooled HRs were 1.97 (univariate: 95% CI, 1.19–3.24; *p* = 0.008; heterogeneity: *Q* = 26.55, *I* ^2^ = 81.2%, *p* < 0.001, **Figure 2A**) and 2.23 (multivariate: 95% CI, 1.29–3.86; *p* = 0.004; heterogeneity: *Q* = 1.14, *I* ^2^ = 12.1%, *p* = 0.286, **Figure 2B**). Five studies reported OS with univariate analysis, and three studies reported OS with multivariate analysis. The pooled HRs for OS were 2.03 (univariate: 95% CI, 1.07–3.84; *p* = 0.029; heterogeneity: *Q* = 27.12, *I* ^2^ = 85.3%, *p* < 0.001, **Figure 3A**) and 1.70 (multivariate: 95% CI, 1.14–2.52; *p* = 0.009; heterogeneity: *Q* = 0.70, *I* ^2^ = 0.0%, *p* = 0.706, **Figure 3B**).

### 3.5 Sensitivity analysis

**Figure 4:**
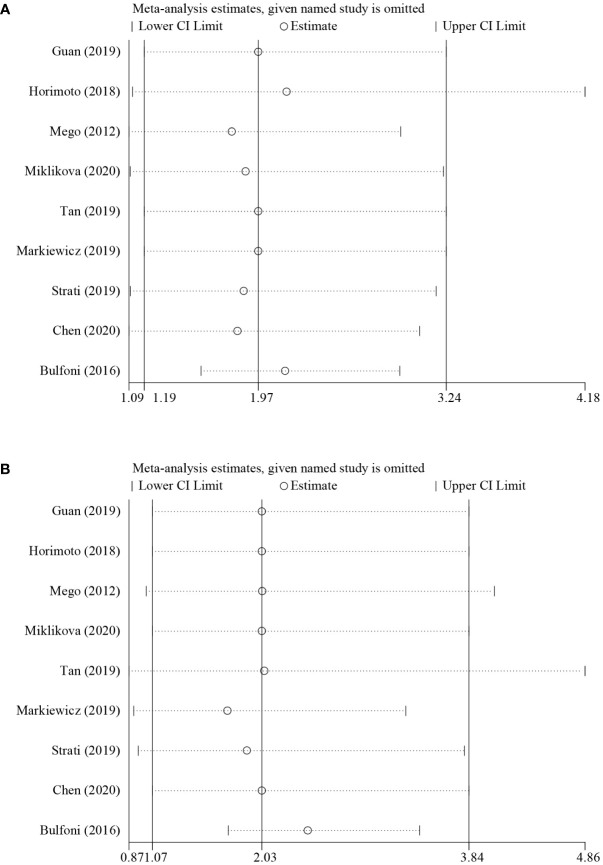
Sensitivity analysis of the pooled HRs for **(A)** PFS and **(B)** OS.

Sensitivity analysis was performed to evaluate the origin of the heterogeneity by removing studies one by one and is shown in **Figure 4**. For the results about PFS (univariate group, **Figure 4A**), although no studies changed the overall trend, an apparent change could be seen when the study of Bulfoni et al. was removed. Moreover, when removing the study of Tan et al., Markiewicz et al., or Strati et al., the pooled estimate of OS (univariate group, **Figure 4B**) showed inconsistencies with others. Considering that only five studies were involved in the analysis, the remaining studies might contribute to the greater heterogeneity. Hence, it seemed that the heterogeneity came mostly from the study of Bulfoni and colleagues.

### 3.6 Subgroup analysis

Because the survival data extracted from the multivariate groups were too few to be further analyzed, the univariate groups were used for subgroup analysis to evaluate the possible correlation with survival outcome.

#### 3.6.1 Cancer stage

**Figure 5:**
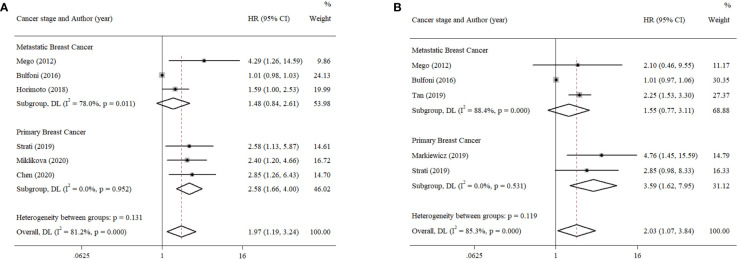
Subgroup analysis of the prognostic value of EMT-CTCs in different breast cancer stages. **(A)** Pooled HR for PFS and **(B)** pooled HR for OS.

The patients in the eligible articles were divided into a metastatic breast cancer group and a primary breast cancer group to evaluate the effect of cancer stage on the prognostic value of EMT-CTCs. The EMT-CTCs from patients with primary breast cancer were significantly associated with poorer PFS (pooled HR = 2.58, 95% CI, 1.66–4.00; *p* < 0.001, **Figure 5A**) and OS (pooled HR = 3.59, 95% CI, 1.62–7.95; *p* = 0.002, **Figure 5B**).

#### 3.6.2 Identification methods for EMT-CTCs

**Figure 6:**
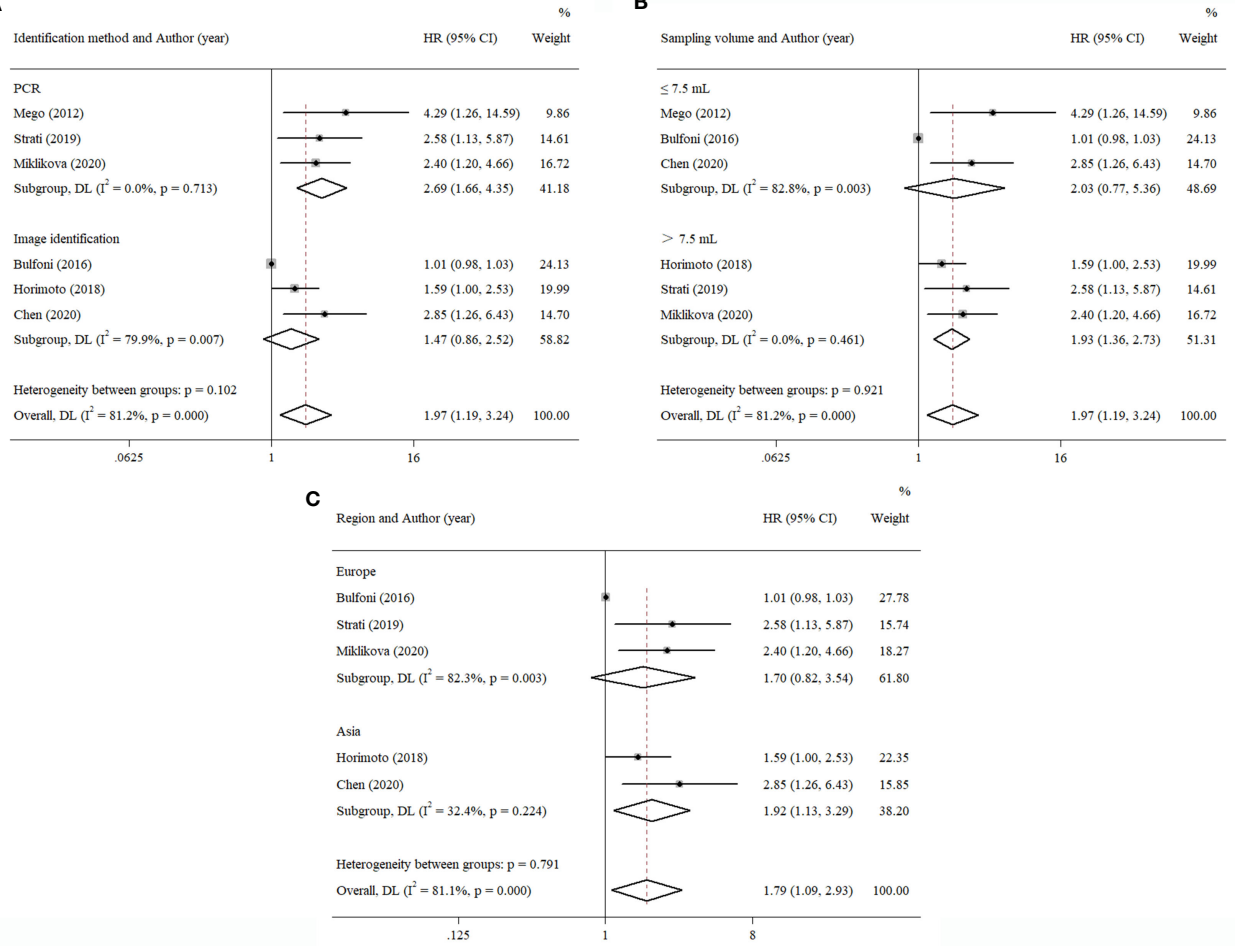
Subgroup analysis of the prognostic value of EMT-CTCs with different **(A)** identification methods, **(B)** sampling volumes, and **(C)** regions.

Compared to the PCR method that characterized the level of EMT-related gene expression, multi-RNA-ISH and immunostaining could be classified into one group, because both of them had to distinguish CTCs by image screening. Thus, the data were separated into the PCR group and the image identification group to explore divergence in survival outcome and are presented in **Figure 6A**. The results showed that the PFS of pooled HR in the PCR group was 2.69 (95% CI, 1.66–4.35; *p* < 0.001), compared to 1.47 (95% CI, 0.86–2.52; *p* = 0.157) in another group.

#### 3.6.3 Sampling volume

We also estimated the effect of sampling volume on the prognostic value of EMT-CTCs (**Figure 6B**). The result showed that pooled HRs of PFS were 1.93 (>7.5 ml, 95% CI, 1.36–2.73; *p* < 0.001) and 2.03 (≤7.5 ml, 95% CI, 0.77–5.36; *p* = 0.151). It implied that a higher sampling volume could more easily determine the prognostic value of EMT-CTCs with the current technical tools.

#### 3.6.4 Region

Except for one study reported in North America, others could be separated into two subgroups: Asia and Europe. The result is shown in **Figure 6C**. The pooled HR of PFS in the Asia group was 1.92 (95% CI, 1.13–3.29; *p* = 0.017). The pooled HR of PFS in the Europe group was 1.70 (95% CI, 0.82–3.54; *p* = 0.154). This result showed that heterogeneity was found in different regions.

### 3.7 Publication bias

**Figure 7:**
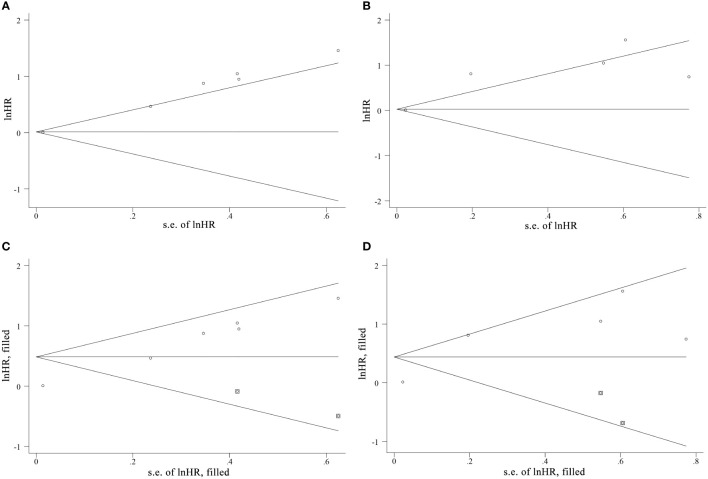
The funnel plots of Begg’s test and the trim-and-fill method of publication bias analysis for **(A, C)** PFS and **(B, D)** OS.

Due to the insufficient data of the multivariate groups, publication bias was only evaluated in the univariate groups. The Begg’s test indicated publication bias among the included studies, as shown in **Figure 7** and confirmed by Egger’s test (PFS: *p* < 0.001, OS: *p* = 0.046). Afterwards, the trim-and-fill method was used to assess the stability of the pooled HRs with a random-effects model. For PFS, the results of pooled HRs remained stable after studies filled (*p* = 0.008 vs. *p* = 0.021), but an obvious change was observed between the pooled HRs of OS (*p* = 0.029 vs. *p* = 0.101).

## 4 Discussion

This meta-analysis revealed that patients with more EMT-CTCs had worse OS and PFS in breast cancer. The result of the subgroup analysis showed that EMT-CTCs could predict more risk of death or tumor progression in the primary breast cancer group, whereas it was not found in the metastatic breast cancer group. In addition, the association between EMT-CTCs and worse survival outcome depended on the method of sorting of CTC phenotypes and the sampling volume at baseline. Inconsistent conclusions were also obtained among patients from different regions. Sensitivity analysis showed heterogeneity from Bulfoni et al.’s study. In this study, the EMT-CTC fraction should reach at least the 50th percentile over all CTCs. This strict criterion might lead to bias when combined with other estimations.

CTCs were considered as a promising tool to understand cancer progression, and their prognostic value has been established in many epithelial cancers (31). In breast cancer, Cristofanilli et al. showed that the presence of ≥5 CTCs per 7.5 ml of blood was related to the shorter PFS and OS in patients with metastatic breast cancer (32). In another retrospective study, a pooled analysis including 2,436 metastatic breast cancer patients showed the same threshold for predicting poorer OS and stratifying metastatic breast cancer patients in stage IV disease (33). As an important signaling pathway for regulating cell migration, EMT was also considered as a critical step in cancer metastasis (34). The mesenchymal feature was identified as the property that would promote mobility, invasion, and resistance to apoptotic stimuli of CTCs (10).

Otherwise, it was reported that the EMT status of primary tumor tissue had a prognostic value (35). However, controversial conclusions were obtained in EMT-CTCs. Mego and colleagues studied 427 patients with primary breast cancer and found that EMT-CTCs were associated with poor prognosis (36). However, Bulfoni et al. found that all phenotype counts of CTCs did not have a prognostic value. When the proportion of hybrid or mesenchymal CTCs exceeded 70% in all CD45-negative cells, a low risk could be observed (30). Moreover, Kasimir-Bauer’s group showed that the presence of CTCs, EMT, and ALDH1 expression was not correlated to any of the prognostic clinical markers (37). Ito et al. presented that epithelial CTCs appeared to be more important determinants of OS than EMT-CTCs (38). However, the group of Markiewicz showed the opposite result (27). In the mouse model, the results showed that epithelial-type systemic breast carcinoma cells with a restricted mesenchymal transition have the strongest lung metastasis ability (39). More metastatic properties could be observed in these hybrid subjects (40, 41).

The abovementioned findings weaken the accuracy and clinical application value of CTC enumeration (42). This might be attributed to the great heterogenicity of CTCs. In recent years, advancing materials and processing technologies facilitated CTC enumeration. However, the CellSearch system remained the only method that the FDA (U.S. Food and Drug Administration) approved. Epithelial cell adhesion molecule (EpCAM), a classical transmembrane protein of CTCs, was still frequently utilized in the capture and isolation step, but during the EMT procedure, EpCAM would be downregulated in the mesenchymal CTCs and the hybrid ones. For immunocytochemical related methods, there was a need to utilize at least two different special targets for CTC identification. Other than EpCAM, Vimentin was commonly used for mesenchymal CTC isolation (43). Identification of all CTC subpopulations by one appropriate biomarker is not possible currently, leading to a lack of comparable standardization method among studies and an unavoidable missing event in the isolation step. To avoid this situation, PCR-related methods could be chosen. Our results also showed the prognostic value of EMT-CTCs in the PCR identification group. The expression of specific genes might lead to a better opportunity to identify the presence of EMT-CTCs. Several EMT-inducing transcription factors were commonly utilized, such as Twist1, Snail, and Slug. Some transcription factors, such as PLS3, were found to be expressed throughout the entire CTC stage (44). However, compared to blood cells, CTCs were too rare to avoid background interference and the whole expression level of transcripts might not accurately depict the variation tendency when EMT occurred. As for PLS3, there were still differences between epithelial CTCs and EMT-CTCs in terms of expression levels (45). Furthermore, the stemness features, which were acquired following the activation of an EMT program (46) and co-expressed with EMT features, would further complicate the results. In some studies, combinations of different markers could even divide CTCs into 17 phenotypes (47). However, few designs included all these effective factors, which should be supported by a detailed and multicentered observational project. For instance, most studies did not consider the possible effect of cluster CTCs, which would survive longer than a single cell and act as a metastasis complex in peripheral blood. Polioudaki’s group reported that cluster CTCs at baseline were associated with poor survival outcome and mesenchymal CTC clusters could independently predict increasing risk of death (48). Hence, it could be anticipated that this situation might not change until the detection strategies were further improved.

As mentioned above, EMT played an essential role in the procedure of metastasis and colonization at distant sites in cancer (49). Increased EMT-CTCs might mean greater risk of metastasis and recurrence. Interestingly, we found that its prognostic value was more significant in the primary cancer group, which might be due to the drug resistance property that allowed the survival of EMT-CTCs after several lines of treatment and reduced their significance among patients with metastatic breast cancer. In a previous study, EMT-CTCs were associated with clinical response to therapy and disease progression (50). Additionally, Tan et al. found that the CTC-specific miR-106b was correlated with EMT status in CTCs and acted as an independent factor for predicting OS (26). This miRNA might lead to the promotion of chemoresistance and EMT processes (51, 52). Mego et al.’s study showed that the prognostic value of EMT-CTCs was more significant in both HR-positive and HER2-negative patients. The authors deduced that the treatment strategy for HER2-positive patients reduced the enrichment of these cells, which resulted in the lack of relationship to prognosis (36). In addition, the stemness feature was also related to treatment resistance. EMT-CTCs’ co-expression with the stemness feature might be harder to eliminate by the therapeutic drug. Papadaki et al. found that the CTCs co-expressed with stemness and EMT features were associated with a worse outcome, whereas the epithelial subtypes did not have any prognostic value (53). Their article implied that the phenotype of co-expressed stemness and EMT features was the most common subtype in patients with metastatic breast cancer. This phenotype was resistant to conventional chemotherapeutic drugs and was found at the primary site (54). The same deduction was discussed by Polioudaki et al. (48). In addition, Miklikova et al. observed an association between adverse outcomes and elevated monocyte-to-lymphocyte ratio in EMT-CTC-positive patients, which implied that inflammation factors might affect the predication value of EMT-CTCs. The report of Guan’s group pointed out that higher enumeration of CTC count was related to more CTC–white blood cell clusters, especially in patients with a high number of EMT-CTCs (55). This result implied that the interaction between EMT-CTCs and some inflammation factors might further affect the prognostic value. It was reported that clusters assembled with CTCs/neutrophils had a higher metastasis-forming potential than those that were not (56). In addition, it was deduced that the dual-positive circulating cells, characterized by the features of epithelial and leukocyte markers co-expression, endowed CTCs with increased motility, invasion, and higher efficiency in metastasis (57). Thus far, the relationship between EMT-CTCs and inflammation factors in terms of prognosis has not been characterized clearly.

There were some limitations in this meta-analysis. First of all, the results might be limited because of the insufficient number of patients. Most patients came from studies discussing primary breast cancer, leading to heterogeneity. Second, due to the various stages and treatment strategies of the included patients at baseline, the heterogeneity among patients might cover the real effects of EMT-CTCs for prognosis. Third, there was no unified criterion among the studies since different methods were utilized for EMT-CTC identification, which might be a further cause of heterogeneity in the analysis. In addition, the publication bias could be observed among the studies. The reason might be the lack of negative results. The unstable results of pooled HRs in the trim-and-fill method indicated that the EMT-CTCs might predict the progression of disease, rather than the death of women with breast cancer.

In conclusion, we evaluated the prognostic value of EMT-CTCs in patients with breast cancer in this meta-analysis. Subgroup analysis showed that the prognostic value of EMT-CTCs might be more significant in patients with primary breast cancer than in those with metastatic breast cancer. Similar findings were obtained between the different identification methods for CTC phenotypes sorting, sampling volumes, and regions. PCR-related methods might be inclined to distinguish different subtypes by specific gene expression, and more sampling volume might mean increasing CTC detection rate. It could be seen that great heterogenicity hindered the clinical application of CTC enumeration. To solve this problem, the interdisciplinary combination of medicine and engineering should be improved in the future to meet the challenge of CTC detection and generate more accurate results. Moreover, in light of the obtained results and limitations of this analysis, a large, multicentric observational study with unified standards for CTC detection and phenotype sorting was necessary to acquire an in-depth understanding of the role of CTCs in female breast cancer.

## Data availability statement

The original contributions presented in the study are included in the article/Supplementary Material. Further inquiries can be directed to the corresponding authors.

## Author contributions

QZ, YL, and LM designed the study. QZ, BL, and QG performed the literature search, and extracted and analyzed the data. QZ and BL conducted the statistical analysis and drafted the manuscript. YL and LM were in charge of the coordination and revised the manuscript. All authors contributed to the article and approved the submitted version.

## Funding

This study was supported by the National Natural Science Foundation of China (81902162 and 82172351), the Science and Technology Department of Henan Province (222102310161) and Natural Science Foundation of Henan Province (212300410395).

## Conflict of interest

The authors declare that the research was conducted in the absence of any commercial or financial relationships that could be construed as a potential conflict of interest.

## Publisher’s note

All claims expressed in this article are solely those of the authors and do not necessarily represent those of their affiliated organizations, or those of the publisher, the editors and the reviewers. Any product that may be evaluated in this article, or claim that may be made by its manufacturer, is not guaranteed or endorsed by the publisher.
